# Optimal nano-silica filler concentration to optimize kinetics, rheology and bonding of self-adhesive composites

**DOI:** 10.1038/s41598-026-43290-5

**Published:** 2026-03-09

**Authors:** Miguel Alves, Pedro Pereira, Diana C. Silva, António H. S. Delgado

**Affiliations:** 1 Egas Moniz Center for Interdisciplinary Research (CiiEM), Egas Moniz School of Health & Science, Campus Universitário, Quinta da Granja, Monte de Caparica, 2829-511 Almada, PT Portugal; 2 Centro de Química Estrutural, Institute of Molecular Sciences and Departamento de Engenharia Química, Instituto Superior Técnico, Universidade de Lisboa, 1049-001 Lisbon, Portugal

**Keywords:** Self-adhesive flowable composite, Nanoparticles, Polymerization kinetics, Dentin bond strength, Resin–dentin interface, Rheology, Chemistry, Engineering, Materials science, Nanoscience and technology

## Abstract

To determine how silica nanoparticle (NanoSi) content modulates polymerization kinetics, dentin bonding, and interfacial adaptation in self-adhesive flowable resin composites (SAFRCs). Five UDMA/PPGDMA/10-MDP SAFRCs containing 0, 2.5, 5, 7.5, or 10 wt% NanoSi were compared to a commercial reference (Vertise Flow, VF). Real-time ATR-FTIR provided t_0.5_, Rp_max_, delay time, and DC_max_; viscosity was measured under controlled shear. Shear bond strength (SBS) was tested at 24 h. Masson’s trichrome and environmental SEM (E-SEM) assessed interfaces. Two-way MANOVA examined formulation and exposure time (20 vs 40 s) effects on kinetics; one-way ANOVA analyzed µSBS (ɑ = 0.05). Formulation and time significantly affected the multivariate kinetic response (Wilks’ λ_formulation = 0.1905, *p* = 0.0099; Wilks’ λ_time = 0.4296, *p* = 0.0045), with no interaction. NanoSi_2.5 polymerized fastest (Rp_max_ 3.17 ± 0.43%·s⁻^1^); NanoSi_5 was slowest (1.61 ± 0.26%·s⁻^1^). DC_max_ increased with filler, peaking at NanoSi_10 (78.77 ± 2.64%); extending exposure from 20 to 40 s raised DC_max_ by 10.23 ± 2.16% without altering kinetics. All pastes were shear-thinning, with viscosity rising monotonically with NanoSi. SBS differed among materials (ANOVA *F* = 3.14, *p* = 0.02): VF was highest (8.6 ± 2.2 MPa); NanoSi_0 reached 6.0 ± 1.1 MPa (NS vs VF); NanoSi_2.5–5 clustered lower (~ 3–4 MPa) and NanoSi_7.5 reached the minimum. Trichrome and E-SEM showed thin, continuous interfaces at 0–2.5 wt% and increasing porosity/thin separations at 7.5–10 wt%. Nanosilica altered cure, flow and interfacial quality trade-offs; faster early kinetics and higher final conversion did not translate into higher SBS, emphasizing the need to optimize interfacial wetting/adaptation alongside mechanical parameters in SAFRC design. A formulation window may exist in which flow, cure, and bonding are balanced to enhance the clinical potential of self-adhesive composites.

## Introduction

Successful design of novel resin-based materials hinges fundamentally on the precise balance between components of the organic matrices and the inorganic fillers^1^. At their interface, molecular-level phenomena, which include adsorption, dispersion stability, particle–matrix affinity, and silane functionalization collectively determine macroscopic material properties^2^. These include mechanical strength, long-term susceptibility towards degradation and also adhesive performance, a largely mechanistic parameter, since it depends on interfacial chemistry (e.g., 10-MDP–Ca salt formation), wetting/infiltration of the smear/etched dentin, and shrinkage-stress during cure^3–5^. Self-adhesive flowable resin composites (SAFRCs) represent a niche subclass of resin-based materials in which this organic–inorganic balance is particularly critical. Their formulation combines a low-viscosity resin matrix containing acidic functional monomers with an inorganic filler phase, allowing them to theoretically condition, bond, and restore in a single step^6^. This design eliminates the separate etch–prime–bond sequence, thereby reducing chairside time and procedure sensitivity^7^, and has been commercially available for over 15 years^8^.

However, despite more than a decade of commercial availability, SAFRCs remain chemically under-optimized, and published research continues to document performance inconsistencies^9,10^. Across multiple laboratory investigations, SAFRCs exhibit very low dentin bond strengths, with microtensile values rarely exceeding ~ 10 MPa, alongside poor sealing, and inferior interfacial and marginal quality compared with conventional adhesive–composite systems^9,11,12^. Clinical evidence is limited to short-term trials, often restricted to small, retentive restorations, with no robust data supporting long-term survival^13,14^. These interfacial shortcomings are potentiated by matrix vulnerabilities such as high viscosity^15^, high water sorption and hygroscopic expansion^16–18^ or limited etching efficacy. Methodologically, most studies have evaluated SAFRCs in their commercial form or applied external procedural modifications (e.g., primer application, co-curing^15^ to improve immediate adhesion, with modest and often temporary gains. Other investigations have examined aging behaviour^19^, polymerization kinetics, or functional monomer variations in experimental formulations^20,21^. Critically, no published work has yet systematically varied the concentration of particles in SAFRC filler phases.

In resin-based restorative materials, silica nanoparticle loading is a primary determinant of viscosity, which in turn governs the ability of the resin phase to wet and infiltrate dental substrates. Excessive filler content increases paste viscosity to the point where monomers cannot adequately etch the smear layer and expose/penetrate the collagen network, resulting in minimal to no hybridization and limited bond strengths^15,22^. Conversely, reducing filler loading can improve flow and adaptation, but at the expense of mechanical reinforcement, polymerization shrinkage control, and resistance to water sorption and hygroscopic degradation. This balance is particularly critical for SAFRCs, which must achieve both intimate substrate contact and sufficient bulk stability without the benefit of a separate bonding agent. Therefore, establishing an optimal silica nanoparticle concentration that supports both adhesive infiltration and sufficient material stability is an essential and remains unexplored. In response, this research reframes the optimization of SAFRCs from an exclusively dental or clinical concern into a fundamental physicochemical and interface chemistry problem.

This study is guided by the hypothesis that optimal silica nanoparticle concentrations can significantly impact interfacial properties and overall adhesive performance by actively modulating physicochemical conditions at the resin-dentin interface.

## Experimental

### Materials and formulation of experimental composites

Briefly, five distinct experimental SAFRCs were developed, with varying concentrations of silica nanoparticles (0, 2.5, 5, 7.5 and 10 wt%), combined with a silanized hybrid filler phase (treated with γ-MPTS at 6%), of barium aluminosilicate 7 µm and 0.7 µm particles, dispersed in a UDMA-PPGDMA flowable organic matrix with 10-MDP added as a functional monomer. The initiator was 1% camphorquinone. The liquid phase was added to the powder, and both were mixed using a centrifugal force mixer (Flacktek Speed Mixer, Landrum, South Carolina) for 45 s at 1250 rpm**.** Materials used consisted of urethane dimethacrylate (UDMA) (Sigma-Aldrich, Schnelldorf, Germany), polypropylene glycol dimethacrylate (PPGDMA) (Sigma-Aldrich, Schnelldorf, Germany), 10-methacryloyloxydecyl dihydrogen phosphate (10-MDP) (DM Healthcare, San Diego, CA, USA), 2-hydroxyethyl methacrylate (HEMA) (Tokyo Chemical Industry, Tokyo, Japan) and camphorquinone (CQ) (PCM Products GmbH Krefeld, Germany). The silica nanoparticles used (AEROSIL® 50) have a specified surface area of 50 m^2^/g and an average particle size of ~ 40 nm (Evonik Operations GmbH, Essen, Germany) and were unsilanized. Silica was introduced as a controlled fraction of the filler phase (0–10 wt% of the inorganic filler phase), replacing an equivalent wt% of Ba glass (ranging from 100 to 90 wt% Ba glass across formulations), to isolate nanosilica-driven changes in rheology, optics (controlling polymerization and interfacial adaptation, while maintaining the same base reinforcing filler chemistry. This corresponded to a constant total filler loading of 71.4 wt% in the final pastes (for NanoSi, this was equivalent to 0.0–7.14 wt% of the whole composite, replacing an equal mass of Ba glass). Backscattered-electron SEM (BSE-SEM) imaging was performed on polished cross-sections at 100 × using a BSD (full) detector at 10 kV (scale bar 300 µm) to qualitatively assess filler dispersion.

The detailed composition of each material is summarized in Table 1. Vertise Flow (Kerr, Orange, CA, USA) was used as the commercial reference material.

**Table 1:** Chemical composition (wt%) of the experimental self-adhesive flowable resin composites (SAFRCs) and commercial control.

Formulation	Organic matrix (wt% of the total organic phase)	Functional monomer (wt%)	Initiator (wt%)	Fillers (wt% of the total filler phase)	Type
NANOSI_0	UDMA (45–55), PPGDMA (20–30), HEMA (3–5)	10-MDP (15–25)	CQ (1%)	Ba glass (100)	Experimental
NANOSI_2.5				Ba glass (97.5) + NanoSi (2.5)	
NANOSI_5				Ba glass (95) + NanoSi (5)	
NANOSI_7.5				Ba glass (92.5) + NanoSi (7.5)	
NANOSI_10				Ba glass (90) + NanoSi (10)	
VERTISE FLOW (CONTROL)	Bis-GMA (N/A), UDMA (5–10) HEMA (5–10)	GPDM (1–5)	CQ/amine	~ 70 vol% glass + ytterbium fluoride	Commercial (Kerr, Orange, CA, USA; shade: A2; batch number: 11883020)

UDMA: urethane dimethacrylate; PPGDMA: polypropylene glycol dimethacrylate; 10-MDP: 10-methacryloyloxydecyl dihydrogen phosphate; HEMA: 2-hydroxyethyl methacrylate; CQ: camphorquinone; Bis-GMA: bisphenol A-glycidyl dimethacrylate; GPDM: glycerol phosphate dimethacrylate; Ba: barium aluminosilicate; NanoSi: silica nanoparticles. Manufacturer data for Vertise Flow based on technical datasheet.

No external tertiary amine co-initiator was added to this camphorquinone-based system. This choice was made because UDMA-based monomers can participate as hydrogen/electron donors for excited camphorquinone, enabling radical generation through hydrogen abstraction from the urethane/carbamate environment of UDMA^21^. In addition, in acidic self-adhesive systems containing 10-MDP, tertiary amines may be protonated by acidic monomers, reducing co-initiator efficiency and introducing acid–base incompatibility as a confounding variable.

### Polymerization kinetics (ATR‑FTIR)

Polymerization kinetics were evaluated using attenuated total reflectance Fourier transform infrared spectroscopy (ATR-FTIR; Spectrum 65, Perkin-Elmer, MA, USA). Disc-shaped specimens (2 mm thickness; *n* = 3 per group) of each experimental SAFRC and the commercial control (Vertise Flow) were fabricated in PTFE molds. Photopolymerization was undertaken with an LED light-curing unit (Elipar™ DeepCure-S, 3 M, St. Paul, MN, USA). The light tip was positioned at 0 mm working distance, against an acetate barrier placed over the specimen surface to standardize the geometry and avoid direct contact. Irradiance was verified with a checkUP™ radiometer (BlueLight Analytics, Halifax, Canada) at the beginning of each session; three consecutive readings were averaged. Under this configuration, irradiance achieved a mean of 1100 mW/cm^2^. Two exposure times were used (20 s and 40 s), corresponding to an estimated radiant exposure at the specimen plane of approximately 22 J/cm^2^ and 44 J/cm^2^, respectively (E ≈ I × t). The curing unit emission was within the blue range (manufacturer-reported peak wavelength; nominal 430–480 nm).

Real-time ATR-FTIR spectra were continuously collected from 700 to 4000 cm^−1^ with a resolution of 4 cm^−1^ for 20 min at 22 ± 2 °C. The degree of conversion (DC) was calculated from the subtraction of the C–O stretching vibration band at 1320 cm^−1^, using the 1336 cm^−1^ as the unchanging standard, according to Delgado et al. (2021). In order to calculate the DC at time t, the following Eq. (1) was used,

1 $${D}_{C} \left(\%\right)=[1-\left(\frac{{A}_{t}}{{A}_{0}}\right)]*100$$

where A_0_ and A_t_ are the C–O stretch absorbance at 1320 cm^−1^ above background level at 1345 cm^−1^ initially and at time t after start of polymerization. Using the 1320 cm⁻^1^ band, rather than the traditional 1640 cm⁻^1^ C=C peak—avoids systematic errors previously detailed (see ref^23^). Continuous acquisition without removing the sample from the crystal eliminates the need for an internal reference peak.

From the DC–time curve, DC_max_ and kinetic parameters were extracted:

- Maximum polymerization rate (Rp_max_, % s⁻^1^)—first derivative peak of DC(%)/time.
- Half-time (*t*_*0.5*_, s)—time to reach 50% of DC_max_.

Plots of conversion (DC) versus 1/t were essentially linear (R^2^ ≈ 1). Since 1/0 → ∞, extrapolating the straight line fitted to the late-time data to 1/t → 0 (i.e., t → ∞) gives the y-intercept, which provides a reliable estimate of the final conversion, DC_max_, as previously undertaken^21^.

### Viscosity measurements

Regarding the rheological properties of SAFRCs, the protocol described by Ferreira et al. (2023)^20^, with previous formulations was used; Rheology assays were performed in triplicate*,* in a rheometer (MCR 92, RhemCompass™ software (Anton Paar, Virginia, USA) using a cone-plate geometry (CP50). The viscosity was measured as a function of shear rate, within the range of 0.1–100 s^−1^, at room temperature and in dark conditions, to avoid pre-polymerization.

### Shear bond strength (SBS)

Regarding the bond strength screening, mid-coronal dentin discs were obtained from cross-sections of 30 sound human molars (Accutom-50, Struers, Denmark), recently extracted (< 3 months) and obtained through the Biobank of Egas Moniz, as approved by the Ethics Committee Board of Egas Moniz School of Health and Science (under process no. EM830). Each dentin disc was submitted to smear layer simulation using a 600-grit SiC under water for 60 s (Struers Polishing, Struers Ballerup, Denmark). A custom CAD/CAM polyacetal mold with three cylindrical wells (internal Ø 1 mm; height 2 mm) was positioned on each dentin disc and the composites were placed to fill each hollow cylinder. Specimens were light-cured in two 20 s exposures (total 40 s) at 0 mm distance, using acetate sheets as stops to stabilize the tip; reported tip irradiance was ≈ 950 mW/cm^2^ (420–480 nm). Molds were removed after curing, and discs were stored in distilled water at 37 °C for 24 h. Each disc (*n* = 5) was then mounted in a universal testing machine (Autograph AG–X, Shimadzu Corporation, Tokyo, Japan). A pre-formed 0.010 orthodontic wire loop was placed flush around each cylinder, aligned parallel to the composite–dentin interface. Load was applied at 1 mm/min until failure; three cylinders per disc were tested.

Shear bond strength was calculated as τ = F/A = F/(π x r^2^) = 4F/(π x d^2^), where F is the load at failure in newtons and r or d are the bonded cylinder’s radius or diameter in millimetres; with F in N and r or d in mm, the result is given in MPa.

### Masson’s trichrome staining of the resin–dentin interface

To qualitatively characterize the resin–dentin interface, bonded tooth specimens restored with SAFRCs were sectioned perpendicular to the bonded surface into ~ 1 mm slices, subsequently ground and polished to ~ 100 µm thickness under water cooling, as described in previous investigations^15,24^. Sections were optionally fixed in 10% neutral-buffered formalin to preserve collagen fibrils prior to staining^24^.

A modified Masson’s trichrome protocol was applied (Table 2), adapted from conventional dental histological methods but excluding mineral oil and introducing a graded ethanol dehydration sequence. This technique allows clear differentiation of interfacial components: red staining indicates exposed/denuded collagen, green denotes mineralized dentin, and the resin composite/adhesive appears beige or lightly stained, enabling visualization of hybridization and potential areas of incomplete resin infiltration^15,24,25^.

**Table 2:** Modified Masson’s trichrome staining protocol for resin–dentin interfaces.

Step	Reagent/procedure	Time/conditions
1	Dehydration in ethanol 70%, 90%, 100%	5 min each
2	1% Acid fuchsin in 1% acetic acid	4–5 min
2.1	Rinse in 1% acetic acid	10 s
2.2	Rinse in distilled water	15 s
3	1% Phosphomolybdic acid	40 min
3.1	Rinse in 1% acetic acid	10 s
3.2	Rinse in distilled water	15 s
4	2% Light Green in 1% acetic acid	5 min
4.1	Rinse in 1% acetic acid	30 s
4.2	Rinse in distilled water	60 s
5	Final dehydration: ethanol 70%, 90%, 100%	2 min each
6	Mounting with resinous medium (Entellan)	Permanent fixation (drying)

After staining, specimens were examined under light microscopy (Olympus CX41, Tokyo, Japan) at 100 × magnification, capturing representative photomicrographs for each group.

For standardized visualization of interfacial staining, all images were processed in Fiji/ImageJ (NIH, USA) using a fixed, reproducible workflow. Colour deconvolution (Colour Deconvolution 2 v2.1 plugin) was used with 32-bit absorbance output to separate stain components. Based on the reference image, the deconvolved channel that best isolated the red-stained component at the dentin–material interface was selected and used for all subsequent analyses. Background correction was applied using rolling-ball subtraction with a fixed radius across all images, followed by fixed brightness scaling to prevent per-image auto-adjustment. For presentation, a uniform false-colour lookup table (LUT) was applied to the standardized channel and exported at the same spatial scale for all groups.

### Environmental scanning electron microscopy (E-SEM)

To further assess the ultrastructural quality of the resin–dentin interface, additional specimens were analyzed using E-SEM (*n* = 2). This technique allows imaging under low-vacuum conditions (≈0.3–0.6 torr water vapor pressure), preserving tissue hydration and reducing artefacts associated with desiccation and sputter coating required for conventional SEM^26^.

Sliced specimens as described in 2.4 were mounted uncoated on aluminum stubs and observed in E-SEM mode (E-SEM; FEI Quanta 400 FEG, FEI Company, Hillsboro, OR, USA). Imaging was performed at 10–15 kV using a gaseous secondary electron detector, with magnifications ranging from 100 to 2000 × . This approach preserves the natural appearance of the hydrated collagen network, avoiding collapse of the interfibrillar matrix and providing reliable morphological information on the adhesive interface^26^. E-SEM analysis focused on the continuity of the hybrid layer, the presence and extension of resin tags, and the detection of marginal gaps or voids.

### Statistical analysis

Normality of data distribution and homogeneity of variances were verified using the Shapiro–Wilk and Levene tests, respectively. All statistical analyses were performed with a significance level set at α = 0.05 (OriginPro 2024, OriginLab Corp., USA).

For polymerisation kinetics (ATR-FTIR), the parameters DC_max_, Rp_max_, t_0.5_ and *delay time* were compared among formulations using a two-way MANOVA, with formulation and light-exposure time (20 vs 40 s) as fixed factors. In the absence of a significant formulation × time interaction, effects were interpreted as main effects. Significant MANOVA effects were followed by univariate two-way ANOVAs for each kinetic outcome, and pairwise comparisons among formulations were performed on estimated marginal means averaged across exposure times, using Bonferroni adjustment (α = 0.05).. For rheological behavior, viscosity values obtained at different shear rates were analyzed using a two-way ANOVA (factors: formulation × shear rate), with Tukey’s post hoc test applied for multiple comparisons. For microshear bond strength (µSBS), mean bond strength values were analyzed with a one-way ANOVA (factor: formulation), followed by Tukey’s post hoc test.

## Results

### Polymerization kinetics (ATR-FTIR)

**Fig. 1:**
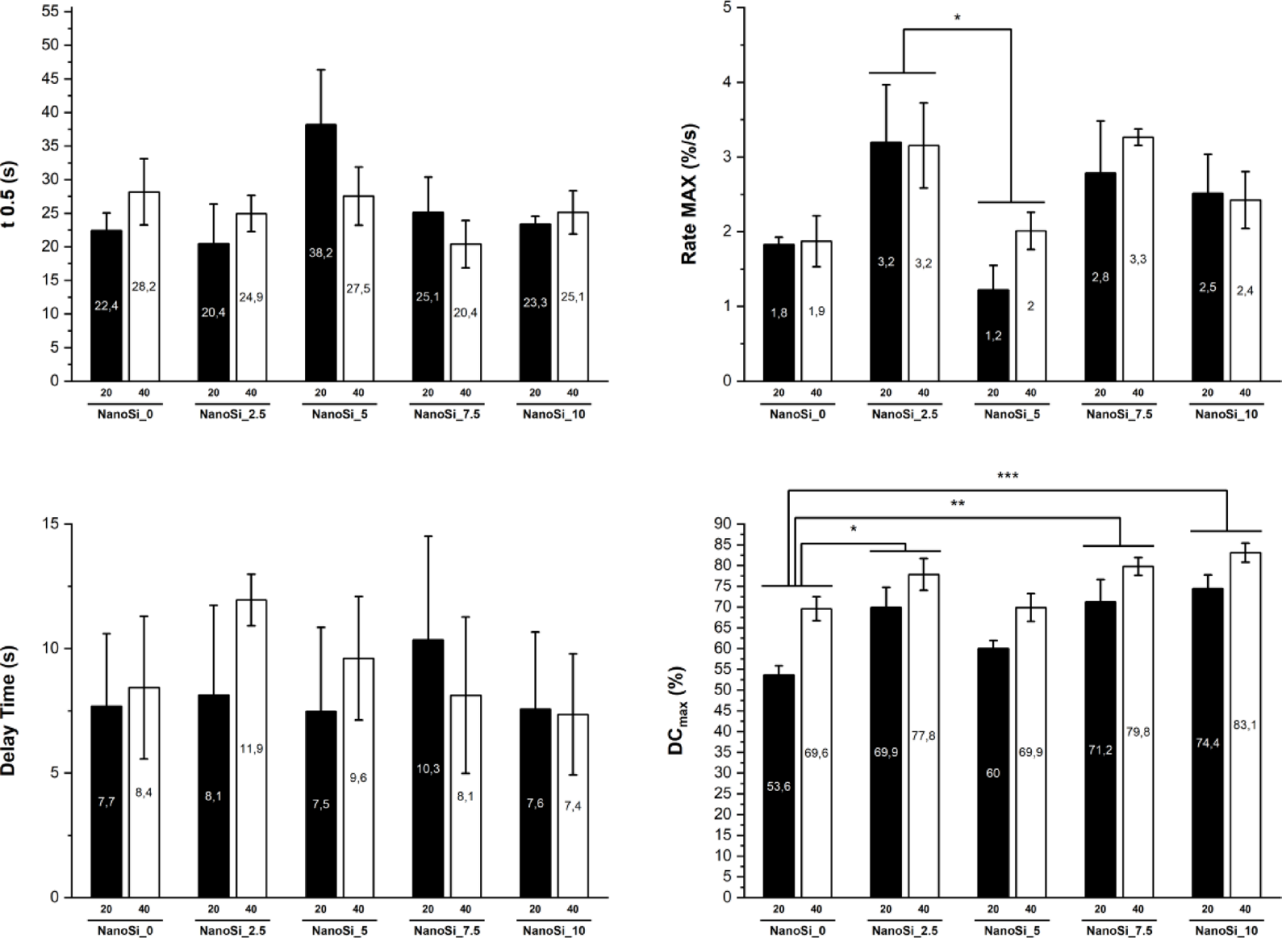
Polymerization kinetics of experimental SAFRCs and the commercial control (Vertise Flow). Mean values (± SE, *n* = 3) are shown for: (**A**) final degree of conversion (DC_max_, %) after 20 s and 40 s of light exposure, (**B**) maximum polymerization rate (Rp_max_, %/s), (**C**) half-time to conversion (t_0.5_, s), and (**D**) delay time (s). Different letters indicate significant differences among formulations (Bonferroni-adjusted pairwise comparisons, α = 0.05; main effect averaged across exposure time).

Polymerization kinetics parameters (t_0.5_, Rp_max_, delay time, DC_max_) are summarized in Fig. 1. A two-way MANOVA (factors: formulation and light-exposure time) performed on the kinetic outcomes—t_0.5_, Rp_max_, delay time, DC_max_—showed a significant main effect of formulation (Wilks’ λ = 0.1905, F(16,52) = 2.368, *p* = 0.009) and of exposure time (Wilks’ λ = 0.4296, F(4,17) = 5.642, *p* = 0.0045). No formulation × time interaction was detected (Wilks’ λ = 0.6586, F≈0.481, *p* = 0.945).

Because the formulation × time interaction was not significant, post hoc comparisons were performed only for the main effect of formulation, using Bonferroni adjustment on estimated marginal means collapsed across exposure time.

Descriptively (mean ± SEM), the shortest half-time to conversion (t₀.₅) was observed for NanoSi_2.5 (22.70 ± 3.08 s) and NanoSi_7.5 (22.74 ± 3.03 s), with NanoSi_10 and NanoSi_0 showing intermediate values (24.24 ± 1.59 s and 25.29 ± 2.81 s, respectively), and NanoSi_5 the slowest (32.86 ± 4.77 s). Peak Rp_max_, followed a similar pattern, being highest for NanoSi_2.5 (3.17 ± 0.43) and NanoSi_7.5 (3.02 ± 0.33), lower for NanoSi_10 (2.47 ± 0.29) and NanoSi_0 (1.85 ± 0.16), and lowest for NanoSi_5 (1.61 ± 0.26). Delay time varied from 7.46 ± 1.76 s (NanoSi_10) to 10.04 ± 1.88 s (NanoSi_2.5) without a clear monotonic trend. The DC_max_ increased with filler content, from 61.6 ± 3.9% (NanoSi_0) to 78.8 ± 2.6% (NanoSi_10); NanoSi_2.5 and NanoSi_7.5 also achieved high conversions (73.9 ± 3.3% and 75.5 ± 3.3%), whereas NanoSi_5 was intermediate (64.9 ± 2.8%). Post-hoc comparisons clarified which contrasts drove these trends. For Rp_max_, NanoSi_2.5 polymerized faster than NanoSi_5 (mean difference = 1.56%/s; Bonferroni-adjusted p = 0.030), while other between-formulation contrasts did not survive adjustment. For DC_max_, both NanoSi_2.5 and NanoSi_7.5 converted more than NanoSi_0 (mean differences =  + 12.27 and + 13.87%-points; Bonferroni-adjusted *p* = 0.018 and *p* = 0.006, respectively); remaining DC_max_ contrasts were not significant after correction.

The main effect of exposure time was limited to conversion: pairwise tests showed no effect of extending curing from 20 to 40 s on t₀.₅, Rp_max_, or delay time, indicating that the significant multivariate “time” effect was driven by increased DC_max_ at longer exposure. Overall, the kinetics point to an optimal window at moderate nanosilica loadings (2.5–7.5 wt%), which accelerated reaction (shorter t₀.₅, higher Rp_max) without compromising initiation, whereas 5 wt% produced the slowest kinetics. At the same time, increasing filler fraction, especially to 7.5–10 wt%, favored higher final conversion compared to lower silica, and longer exposure preferentially enhanced DC_max_.

### Rheological behavior

**Fig. 2:**
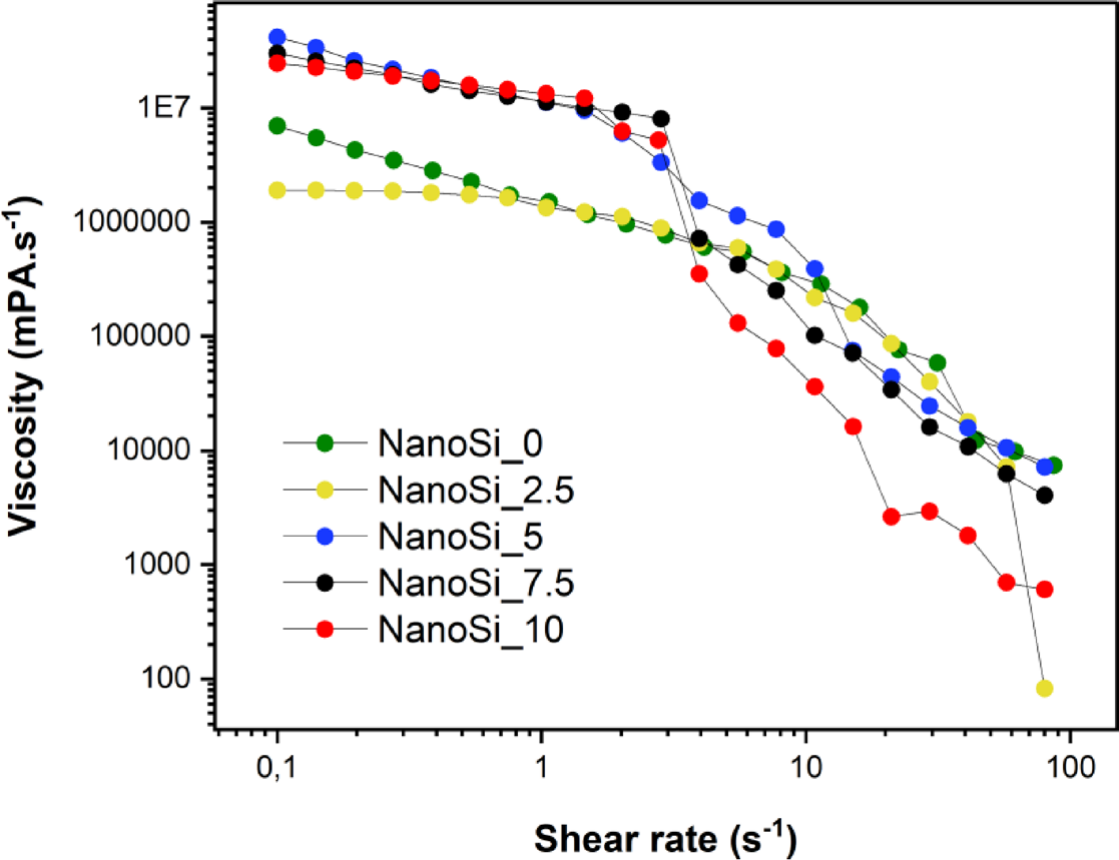
Flow curves of experimental SAFRCs (NanoSi_0, NanoSi_2.5, NanoSi_5, NanoSi_7.5 NanoSi_10). Viscosity (mPa·s) as a function of shear rate (s⁻^1^, log scale). All materials showed shear-thinning behavior, with viscosity increasing in proportion to nanosilica concentration. Data represent mean values* (n* = *3).*

All experimental SAFRCs showed a shear-thinning profile, with viscosity decreasing as the shear rate increased (Fig. 2). Still, clear differences were observed between formulations depending on nanosilica content. NanoSi_2.5 presented the lowest viscosity in the low-shear region (e.g., 0.1 s⁻^1^), whereas NanoSi_5, NanoSi_7.5 and NanoSi_10 exhibited substantially higher viscosities at the same low shear rates. As shear rate increased, the separation between curves narrowed and the rank order changed, with NanoSi_10 showing the lowest viscosities in the intermediate–high shear region. At a reference shear rate of 10 s⁻^1^, the apparent viscosity (mean curve; *n* = 3) was 4.27 × 10^4^ mPa·s for NanoSi_10, 1.24 × 10^5^ mPa·s for NanoSi_7.5, 2.49 × 10^5^ mPa·s for NanoSi_2.5, 3.12 × 10^5^ mPa·s for NanoSi_0, and 4.66 × 10^5^ mPa·s for NanoSi_5, giving the ascending order NanoSi_10 < NanoSi_7.5 < NanoSi_2.5 < NanoSi_0 < NanoSi_5. At the highest shear rates tested (≈80–100 s⁻^1^), viscosities converged toward lower values, consistent with shear-thinning across all formulations.

**Fig. 3:**
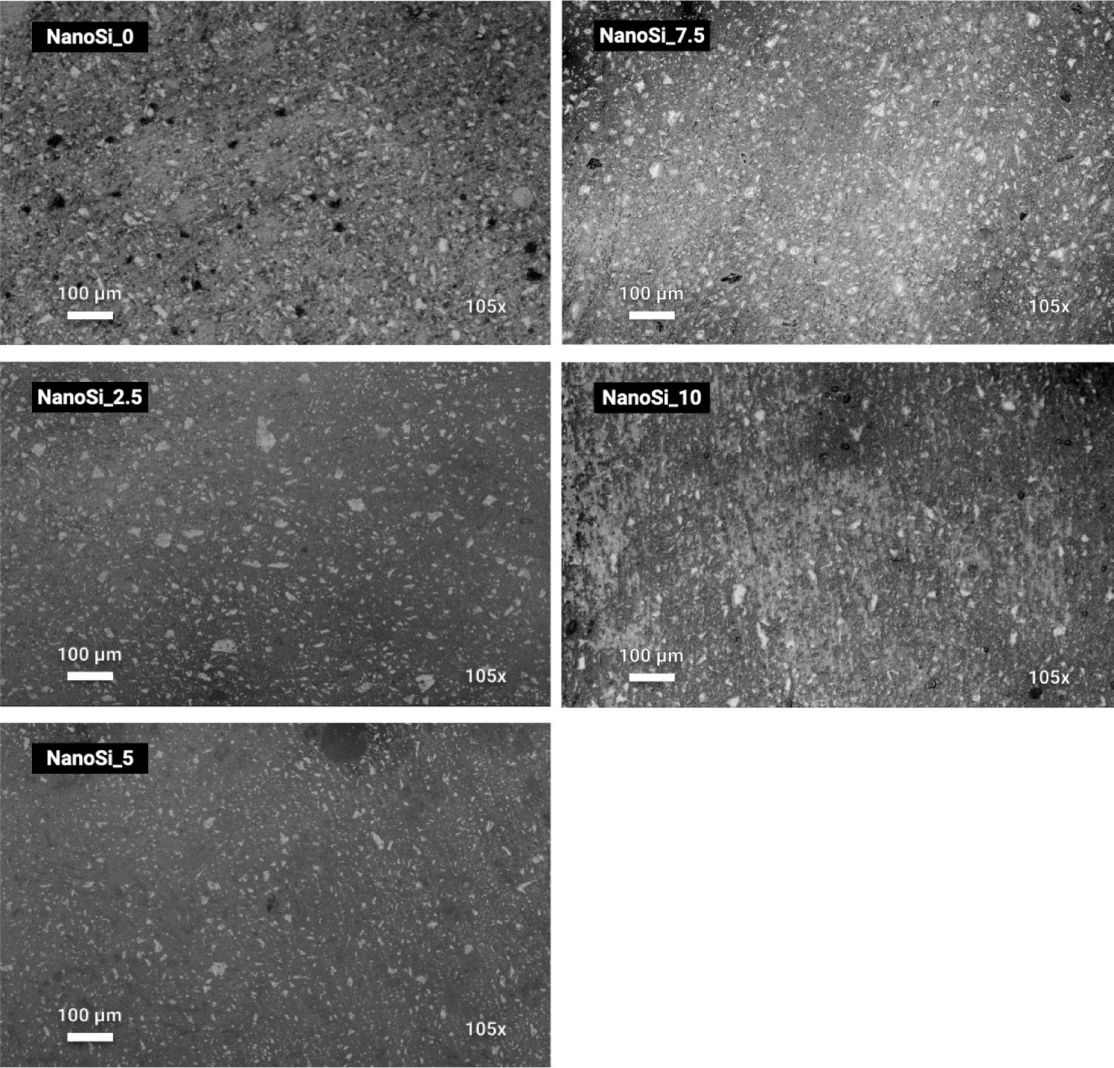
Representative backscattered-electron SEM (BSE-SEM) micrographs of polished cross-sections from the experimental SAFRCs containing 0, 2.5, 5, 7.5, and 10 wt% nanosilica (NanoSi_0–NanoSi_10), illustrating qualitative filler dispersion and the absence of micron-scale agglomerates. Images were acquired at 105 × (scale bar = 100 µm; 10 kV; BSD full).

BSE-SEM confirmed a visually homogeneous filler distribution without large agglomerates at the scale examined (Fig. 3), supporting that the rheology/kinetic differences reflect formulation effects rather than gross mixing defects.

### Shear bond strength

**Fig. 4:**
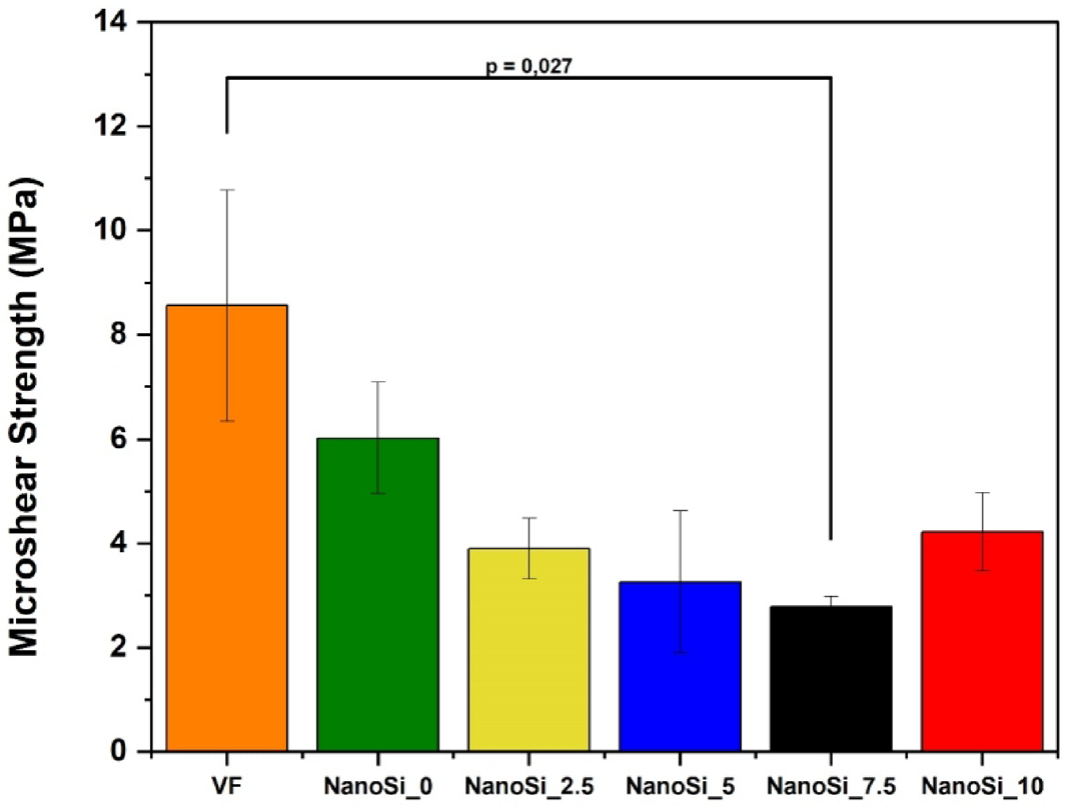
Microshear bond strength (µSBS, MPa) of experimental SAFRCs with increasing nanosilica concentrations (0–10 wt%) and the commercial control Vertise Flow. Bars represent mean ± SD (*n* = 5). One-way ANOVA followed by Tukey’s test (α = 0.05). Significant differences were detected only between Vertise Flow and NanoSi_7.5.

Shear bond strength results are summarized in Fig. 4. The commercial control Vertise Flow (VF) presented the highest mean values (8.6 ± 2.2 MPa), while the experimental formulation NanoSi_0 achieved mean values of 6.0 ± 1.1 MPa. The overall ANOVA confirmed that µSBS varied according to the composite used (ANOVA one-way, F = 3.14; *p* = 0.02).

The intermediate formulations NanoSi_2.5 and NanoSi_5 showed lower bond strength values (~ 3.3–4 MPa), clustering together with no significant differences from NanoSi_0. NanoSi_7.5 exhibited the lowest mean bond strength (2.8 ± 0.2 MPa) and was the only group that differed significantly from VF (*p* < 0.05). Finally, NanoSi_10 displayed a partial recovery in bond strength (4.2 ± 0.8 MPa), with values higher than NanoSi_7.5 but still not statistically different from the other experimental formulations.

Failure modes can be seen in the table below (Table 3). Overall, high PTF prevalence was observed in the higher-NanoSi groups (≥ 2.5 wt%), consistent with reduced bond integrity.

**Table 3:** Failure mode distribution for microshear bond strength (SBS) specimens.

Material	Adhesive (%)	Mixed (%)	Pre-test (PTF, %)
Vertise Flow	67	27	6
NanoSi_0	60	–	40
NanoSi_2.5	40	–	60
NanoSi_5	30	–	70
NanoSi_7.5	35	–	65
NanoSi_10	35	–	65

### Masson’s trichrome staining

**Fig. 5:**
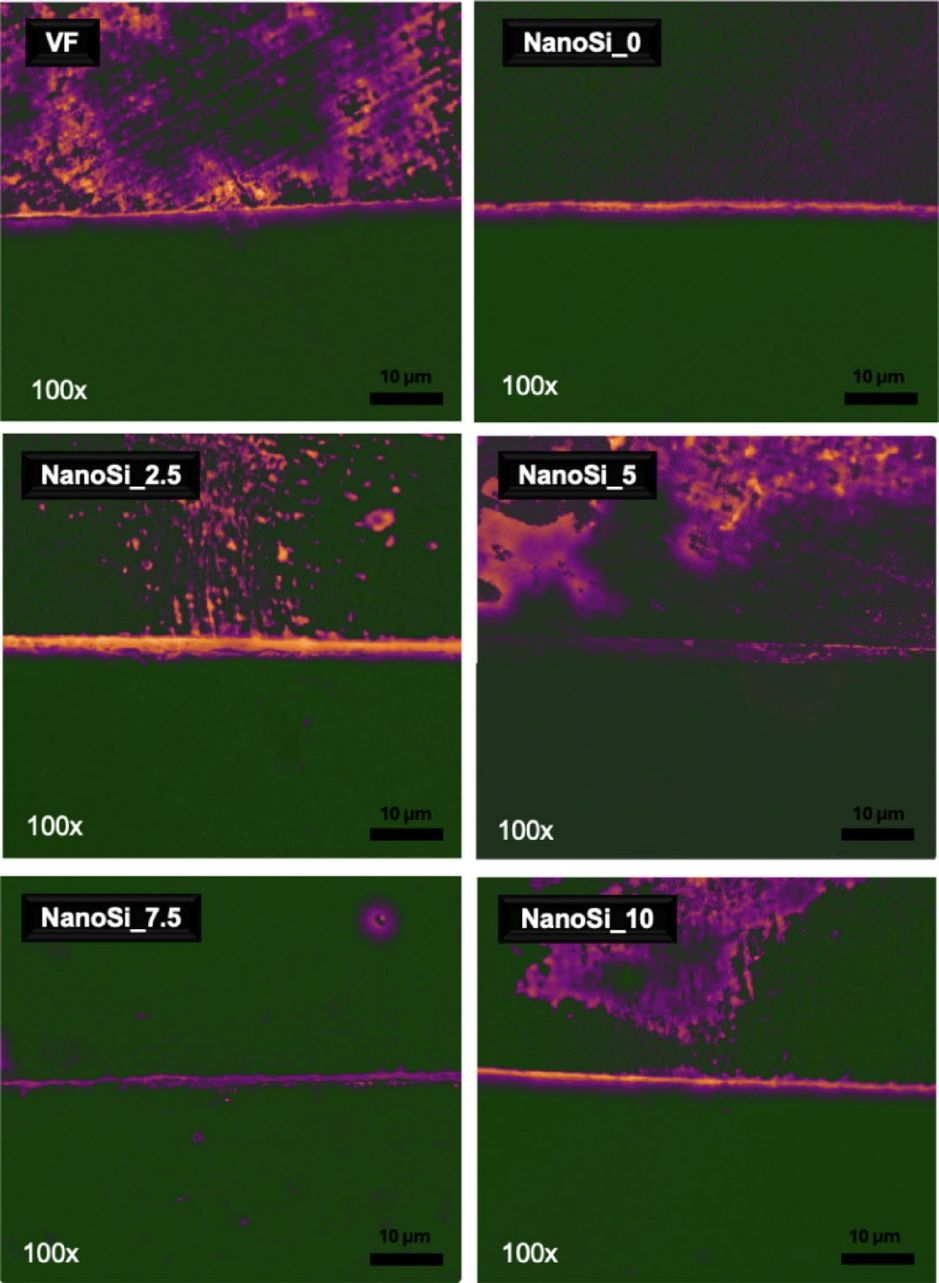
Standardized Masson’s trichrome interface maps (100 × ; scale bar = 10 µm) for the commercial control (VF) and experimental self-adhesive composites containing 0–10 wt% nanosilica (NanoSi_0 to NanoSi_10). Images were processed in Fiji/ImageJ using colour deconvolution and identical display scaling across groups. In the displayed maps, top surface is dentin, middle line is the interface and lower bottom represents the composite. Higher interfacial signal (intense yellow) corresponds to collagen-positive staining at the dentin–composite boundary, consistent with exposed/uninfiltrated collagen (i.e., incomplete collagen hybridization/infiltration at the interface).

Representative photomicrographs of the resin–dentin interfaces stained with Masson’s trichrome are presented in Fig. 5. Polished cross-Sects. (100 × ; 10 µm scale bar) showed a thin, dye-intense band at the dentin–composite boundary in all groups, consistent with a superficial, organic-rich interfacial zone and/or localized dye accumulation. Across formulations, the thickness and continuity of this band and the occurrence of adjacent porosities varied with nanosilica content.

In the commercial control (VF), the interfacial band was conspicuous but irregular, with focal broadening and small dye pools at the composite side of the boundary. The unfilled experimental resin (NanoSi_0) presented the sharpest interface: a narrow, continuous band with evident exposed collagen, and a smooth transition between phases can be seen. Adding 2.5% nanosilica induced the creation of the thickest interdiffusion zone out of all samples, and consequently higher collagen exposure; isolated voids adjacent to the boundary were occasionally observed. At 5% nanosilica, dye-rich areas were very discrete and collagen exposure was limited indicating better infiltration and uniform adaptation. The 7.5% material displayed a continuous boundary, with almost no dyed collagen and some fine speckles/porosities near the interface than at lower filler loads. The 10% nanosilica composite again showed a narrow, sharply delineated band with few defects and a collagen exposure trend mimicking the control group VF and 0% nanosilica. Overall, trichrome staining indicates that moderate nanosilica contents (5–7.5%) yield the most regular and continuous interfacial zone, 2.5% produced the most heterogeneous boundary with higher exposure, and higher loadings (10%) yield a thin band closer to the unfilled resin.

### E-SEM analysis

**Fig. 6:**
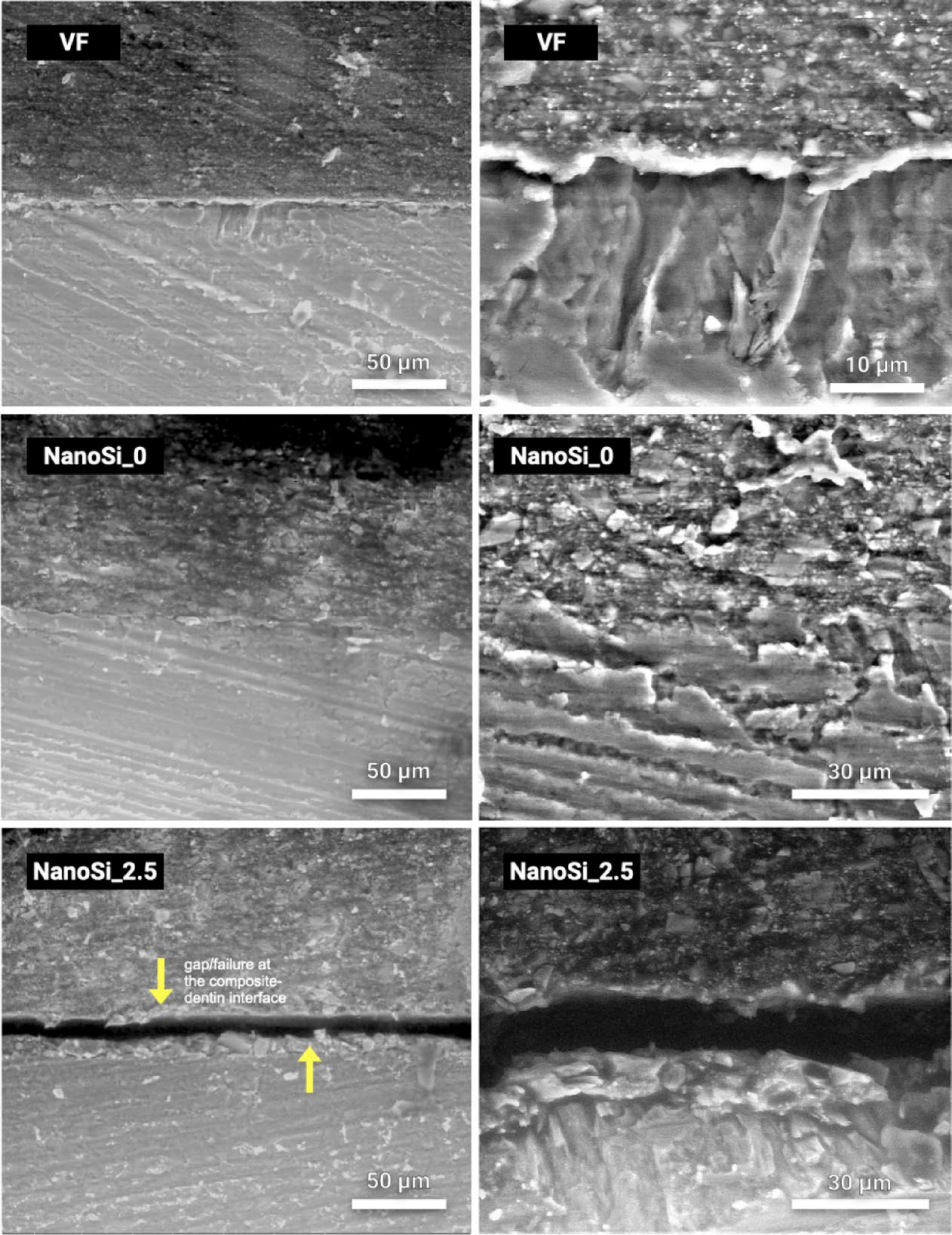
Representative environmental SEM (E-SEM) micrographs of resin–dentin interfaces formed by the commercial self-adhesive flowable composite (Vertise Flow; VF) and experimental SAFRCs containing 0 wt% (NanoSi_0) and 2.5 wt% nanosilica (NanoSi_2.5). For each material, a lower-magnification overview (left; scale bar = 50 µm) and a higher-magnification view of the interface (right; scale bars as indicated) are shown. The composite is located in the upper region and dentin in the lower region. VF and NanoSi_0 show a continuous interfacial contact region without an apparent hybrid layer. In NanoSi_2.5, a linear discontinuity consistent with an interfacial gap is visible along portions of the boundary (arrows).

**Fig. 7:**
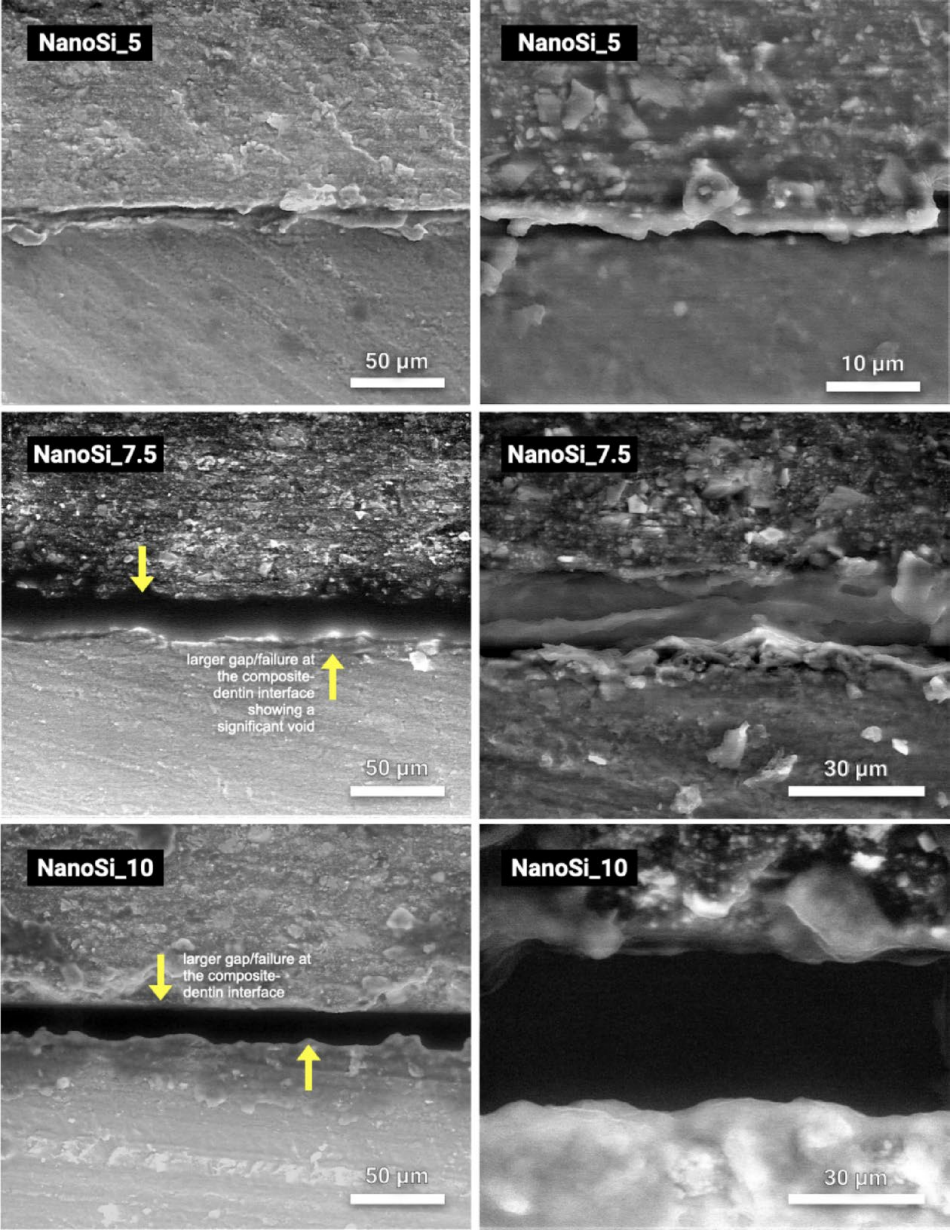
Representative environmental SEM (E-SEM) micrographs of resin–dentin interfaces formed by experimental SAFRCs containing 5 wt% (NanoSi_5), 7.5 wt% (NanoSi_7.5), and 10 wt% nanosilica (NanoSi_10). NanoSi_5 exhibits an irregular boundary with localized separations. NanoSi_7.5 and NanoSi_10 display more pronounced interfacial discontinuities, including extended separation bands and void-like regions (arrows), indicative of incomplete adaptation along the resin-dentin interface.

Representative E-SEM micrographs of the dentin–resin interfaces are shown in Fig. 6 and in Fig. 7, with low and high magnification. Across groups, the dentin surface presented parallel machining marks consistent with a retained smear layer; no classic hybrid layer or long resin tags were discernible in any specimen. In the commercial control (Vertise Flow) exhibited a continuous, well-adapted resin–dentin interface with no evident gap line at the magnification examined. The experimental self-adhesive composite without nanosilica (NanoSi_0) exhibited the most intimate contact: a largely continuous, gap-free interface with a smooth transition from composite to dentin and only sporadic, submicrometric discontinuities, mirroring also the VF group. Contrastingly, the formulation containing 2.5% nanosilica (NanoSi_2.5) showed poorer adaptation, with more frequent interfacial discontinuities and void-like features along the boundary. At 5% nanosilica (NanoSi_5), adaptation was generally continuous; however, shallow “micro-steps” and scattered interfacial voids (≈1–3 µm) appeared intermittently, and small pores were more common within the adjacent composite matrix than in lower-filled groups. The 7.5% and 10% nanosilica formulations (NanoSi_7.5 and NanoSi_10) showed the most pronounced interfacial irregularities: an extended, dark separation line was evident along long segments of the interface, with periodic local detachments and a higher frequency of adjacent composite-side porosities; in NanoSi_10 this separation appeared slightly thicker and more continuous than in NanoSi_7.5.

## Discussion

### Importance of the study and justification

Poor oral health has far-reaching effects beyond the healthcare system, affecting nearly 3.5 billion people worldwide^27^ and imposing an estimated annual burden of $710 billion in treatment costs and productivity losses. The development of SAFRCs represents an attempt to simplify restorative dentistry by combining multiple steps, thereby reducing technique sensitivity, costs and chairside time^13,21,28^. This potentially streamlines treatments and their access. Despite these advantages, current SAFRCs frequently exhibit weaker bonding to dentin, higher microleakage, and poorer long-term stability compared to conventional adhesive systems^9,20,29,30^. The limitations are generally attributed to their high viscosity, limited demineralization potential, and inability to form a well-defined hybrid layer^11,31^.

Recent literature emphasizes the importance of formulation optimization to enhance the performance of SAFRCs^32,33^. In particular, filler loading plays a central role: excessive filler increases viscosity and restricts monomer infiltration into the dentin matrix, while insufficient filler compromises mechanical reinforcement and increases polymerization shrinkage^34,35^.

### Polymerization kinetics and rheology

The kinetic profile of these experimental SAFRCs was governed by a trade-off between early-stage reactivity and ultimate network formation that depended non-monotonically on nanosilica content. The 2.5 wt% formulation reached the highest rate of polymerization (Rp_max_ ≈ 3.17 ± 0.43%·s⁻^1^) with a short half-time (t_0.5_ ≈ 22.70 ± 3.08 s), consistent with reduced viscosity facilitating radical mobility and light transmission^21,36^, whereas 5 wt% depressed the rate and lengthened t_0.5_ (1.61 ± 0.26%·s⁻^1^; 32.86 ± 4.77 s). Yet, final conversion increased with filler content, peaking at 10 wt% (DC_max_ 78.77 ± 2.64%), and was further improved by extending exposure from 20 to 40 s without altering the early-stage metrics. Similar findings were reported by Omrani et al*.*^18^, who observed that self-adhesive composites could achieve high final degrees of conversion but with slower initial kinetics compared to conventional flowables. This pattern is consistent with different mechanisms.

First, submicrometric silica changes the way light is transported through the paste. At low loading (≈2.5 wt%), forward scattering can increase the mean optical path length, enhancing initiator excitation and Rp_max_; at intermediate loading (5 wt%), increased turbidity and/or incipient agglomeration are more likely to attenuate photon flux locally, slowing the network build-up; and at higher loading (≥ 7.5 wt%), improved index matching by the maturing matrix and a denser scattering field can partly restore light delivery during the exposure window, allowing conversions to catch up despite only moderate rates. Because the 40 s exposure increased DC_max_ but did not change Rp_max_, delay time, or t₀.₅, the system appears energy-limited rather than chemically rate-limited under our conditions—extra radiant exposure drives late conversion once vitrification slows propagation. This interpretation aligns with ATR-FTIR kinetics in self-adhesive composites showing that longer exposures predominantly raise DC_max_, while Rp_max_ is comparatively insensitive to exposure time.

Rheological characterization confirmed that all experimental SAFRCs behaved as non-Newtonian, shear-thinning pastes, with viscosity decreasing markedly as shear rate increased. The effect of NanoSi on viscosity was not monotonic across the shear range, indicating that the influence of nanosilica depended on the balance between particle–particle interactions at low shear and structural breakdown under higher shear. The 2.5% nanosilica group exhibited the most stable flow, suggesting optimal dispersion and handling. These observations align with reports that flowable composites with reduced filler exhibit superior adaptability to cavity walls due to enhanced fluidity^37^. However, excessive reduction in filler compromises strength, as highlighted by Mirică et al.^37^, who showed that filler loading is directly proportional to mechanical reinforcement.

### Microshear bond strength

Bond strength results followed an inverse relationship with filler loading. Vertise Flow (control) showed the highest mean µSBS (8.6 ± 2.2 MPa), but this did not differ significantly from NanoSi_0. Intermediate groups (2.5–5%) yielded ~ 4–5 MPa, while NanoSi_7.5 presented the lowest values and was significantly different from the control. These data indicate that the self-adhesive mode is intrinsically weak and that small changes in rheology and interfacial quality, exacerbated here by nanosilica loading, translate directly into bond strength penalties. These findings corroborate systematic reviews reporting that SAFRCs exhibit consistently lower bond strengths compared with conventional adhesive systems^9,10^. The reduced µSBS in highly filled groups reflects their impaired ability to infiltrate dentin, leaving collagen fibrils partially exposed. Similar trends were observed with Ingles et al.^15^, having demonstrated that additional priming improved SAFRC bonding by reducing the presence of unprotected collagen, highlighting the role of infiltration.

Mechanistically, two factors likely governed the downward trend with increasing nanosilica. First, filler addition raises viscosity and reduces the effective concentration and mobility of acidic/functional monomers at the interface, impairing wetting of the smear-covered dentin and limiting micromechanical interlocking. This interpretation aligns with E-SEM and Masson’s trichrome observations showing a largely smear-mediated, hybrid-layer–free interface and thin interfacial discontinuities that were most evident at 7.5–10 wt% nanosilica. Second, the acidic monomer dispersed in higher percentage of inert silica lowers the potential for chemical bonding to hydroxyapatite, an already tenuous mechanism for GPDM-type self-adhesive systems compared with contemporary 10-MDP chemistries^9,10^. The literature consistently shows that self-adhesive flowables, including VF, generate lower dentin bond strengths than conventional adhesives, and that these bonds are highly technique- and test-condition–dependent. Meta-analytic evidence confirms inferior immediate and long-term bonding of self-adhesive flowables to both enamel and dentin relative to conventional adhesive/composite protocols^9^, reinforcing the modest values observed here.

Chemical considerations help explain why the higher percentage silica-filled experimental resins underperform. Stable ionic bonding to hydroxyapatite is monomer-specific: 10-MDP forms low-solubility Ca-MDP salts and can self-assemble in nanolayers, phenomena linked to higher and more durable adhesion; by contrast, other phosphate/acidic monomers (e.g., phenyl-P) show inferior hydrolytic stability. Thus, increasing an inert nanofiller at the expense of available acidic monomer would be expected to further depress chemical interaction and worsen wetting, exactly as seen in the 2.5–7.5 wt% region. The small rebound at 10 wt% may reflect a modest balance between interfacial conversion/shrinkage-stress development and rheology (slower kinetics and higher modulus at cure can sometimes offset very low interfacial integration), but it remained statistically similar to other experimental groups, while still well below VF.

### Masson’s trichrome staining

Histological evaluation reinforced the µSBS findings. NanoSi_0 and NanoSi_2.5 displayed continuous inter-diffusion zones with minimal red staining, likely indicating minimal etching and sufficient collagen encapsulation. In contrast, NanoSi_7.5 and NanoSi_10 showed more prominent red bands at the interface, denoting exposed collagen fibrils. Vertise Flow presented an intermediate pattern, with a shallow interdiffusion zone layer and focal collagen exposure. Previous studies have demonstrated that Masson’s trichrome is effective in highlighting hybrid layer characteristics and the degree of collagen encapsulation at adhesive interfaces^15,38,39^.

These results mirror those reported by Ingles et al.^15^, who observed substantial exposed collagen when SAFRCs were applied without pretreatment, while trichrome staining confirmed improved infiltration when primers were co-cured. Similarly, Hanabusa et al.^11^ demonstrated via TEM that experimental self-adhesive composites often leave a thin demineralized zone with incomplete resin penetration. These findings confirm that viscosity may govern the extent of collagen infiltration, with lower-filler formulations providing better inter-diffusion zones.

### E-SEM interfacial analysis

Finally, E-SEM analysis revealed well-adapted, gap-free interfaces for NanoSi_0 and NanoSi_2.5, while higher filler groups exhibited frequent interfacial gaps and voids. Across all groups, no distinct hybrid layer or resin tags were observed, in agreement with previous microscopy studies showing limited interfacial interaction for SAFRCs^11,31,40^. Voids observed in NanoSi-containing groups are consistent with two coupled phenomena. First, nanosilica increases low-shear viscosity and yield-like behavior, which can reduce interfacial wetting. Second, as the material hardens during cure, limited pre-gel flow/stress relaxation at the interface likely promotes localized debonding under polymerization shrinkage, producing thin separation bands that appear as void-like regions in E-SEM; Interfacial discontinuities also arise when polymerization shrinkage stress develops faster than (or exceeds) the developing tooth–material bond strength^41^. This is particularly relevant in low-bond strength materials such as SAFRCs. The higher prevalence of pre-test failures in the higher-NanoSi groups supports this reduced interfacial integrity.

Spencer et al.^34^ described such morphological deficiencies as the principal weak point of adhesive interfaces, predisposing restorations to hydrolysis and degradation. Our results support this view, as poor adaptation and collagen exposure were evident in high-filler groups. Conversely, lower-filler formulations exhibited improved continuity, suggesting viscosity control is critical for ensuring intimate adaptation. Mechanistically, different effects likely drove the filler-dependent pattern. First, placement rheology: higher nanosilica increases low-shear viscosity, as stated, and reduces capillary penetration into smear micro-porosities, impairing formation of a continuous resin–smear complex^4,42^. Secondly, the added silica can scatter the curing light, altering the local gel point and creating a stiffer, earlier-vitrified interfacial layer that has less ability to flow and relax volumetric shrinkage, predisposing to a thin gap line^42^. Indeed, considering all experimentals, the 2.5 wt% formulation polymerized fastest, whereas 7.5–10 wt% slowed (higher t_0.5_, lower Rp_max_); delayed gelation at low/intermediate filler allows more pre-gel flow and improved adaptation, whereas slower but more heterogeneous curing at high filler traps porosity and accentuates boundary separation^43,44^.

All interfacial findings suggest that nanosilica alters *both* (i) interfacial wetting/penetration through the smear layer and (ii) the shrinkage-stress/gelation balance that governs whether intimate contact is retained during cure. All groups showed a smear-layer–mediated interface without an obvious hybrid layer or resin tags, indicating that bonding was limited by the capacity of the acidic resin to permeate smear micro-porosities rather than by formation of a classic demineralized collagen network. In this context, filler-driven viscosity changes can restrict capillary penetration and reduce interfacial continuity, consistent with the more frequent separation lines and composite-side porosity observed at ≥ 7.5 wt% in E-SEM. The trichrome maps, however, indicate that collagen-positive signal at the boundary (consistent with exposed/uninfiltrated collagen) did not vary monotonically with nanosilica; rather, the lowest collagen signal was observed at intermediate 5–7.5 wt% while 2.5 wt% showed a more heterogeneous collagen-positive interfacial zone.

This divergence suggests that interfacial quality in SAFRCs may be in fact multi-dimensional. Reduced collagen staining does not necessarily guarantee gap-free adaptation, which may be lost during polymerization contraction if flow/relaxation is limited at the gel point. Consistent with this, kinetics were fastest at 2.5 wt% but slower at higher loadings, while DC_max_ increased with filler and with longer exposure, implying that post-gel conversion can be enhanced even when early reaction is compromised.

### Study limitations and future recommendations

Factors such as pulpal pressure, cavity configuration, and moisture variability were not simulated, all of which influence adhesive behaviour and should also be considered. Furthermore, characterization of the filler dispersion, optical transmittance/scattering, or silane coupling chemistry type and concentration, all can influence light transport, viscosity, susceptibility towards hydrolytic degradation and the efficiency of stress transfer at the matrix–filler interface. Dedicated optical and interfacial analyses (e.g., RI matching, agglomerate sizing, silane chemistry), are thus recommended in future studies. Aging assessment were deliberately left out since the aim of this study was an initial rapid screening characterization of incipient formulations which could then be further optimized in coming assays. While nanosilica is often introduced to enhance reinforcement, previous studies indicate that low nano-silica additions may in fact improve mechanical properties, whereas higher loadings can yield mixed outcomes depending on dispersion and interfacial coupling^45^, underscoring the need to directly test mechanical endpoints in future iterations. Mechanical testing is planned in follow-up work, although related UDMA-based self-adhesive/remineralising composites from the same group^21^, have shown that these formulations achieve more than acceptable and ISO compatible flexural strength values. Because familywise-adjusted pairwise testing is conservative in the presence of scatter typical of SBS, smaller differences among experimental formulations should be interpreted using effect sizes and 95% CIs and also confirmed in follow-up studies designed specifically for aging and moderate effect detection.

Overall, the dataset supports a formulation trade-off in which intermediate nanosilica loadings can accelerate early cure while higher loadings favor higher ultimate conversion, whereas bonding and interfacial continuity remain constrained by smear-layer wetting and cure-stress retention rather than by formation of a classic hybrid layer.

## Conclusions

Within the limitations of this laboratory study, nanosilica affected curing, rheology, and interfaces non-monotonically, so no single loading optimized all outcomes. Kinetics were fastest at 2.5 wt% silica, while final conversion rose with filler and peaked at 10 wt. All formulations were shear-thinning, and viscosity rankings depended on shear rate. For bonding, the commercial reference remained highest and the best experimental performance was the unfilled resin. Microscopy showed smear-layer–mediated interfaces without a distinct hybrid layer; higher nanosilica showed more separation lines/voids, and evidence of collagen envelopment suggested better encapsulation at 5–7.5 wt% than at 2.5 wt%. These findings highlight the potential for optimizing filler fraction to advance next-generation SAFRCs toward improved clinical reliability.

## Data Availability

Full data will be made available upon request to the corresponding author.
